# The P1 Protein of *Watermelon mosaic virus* Compromises the Activity as RNA Silencing Suppressor of the P25 Protein of *Cucurbit yellow stunting disorder virus*

**DOI:** 10.3389/fmicb.2021.645530

**Published:** 2021-03-22

**Authors:** Maria Luisa Domingo-Calap, Ornela Chase, Mariona Estapé, Ana Beatriz Moreno, Juan José López-Moya

**Affiliations:** ^1^Centre for Research in Agricultural Genomics (CRAG), CSIC-IRTA-UAB-UB, Campus UAB Bellaterra, Barcelona, Spain; ^2^Instituto Valencia de Investigaciones Agrarias, IVIA, Valencia, Spain; ^3^Universitair Medisch Centrum, UMC, Utrecht, Netherlands; ^4^Consejo Superior de Investigaciones Científicas (CSIC), Barcelona, Spain

**Keywords:** RNA silencing suppression, watermelon mosaic potyvirus, cucurbit yellow stunting disease crinivirus, plant virus mixed infection, virus pathogenesis in plants

## Abstract

Mixed viral infections in plants involving a potyvirus and other unrelated virus often result in synergistic effects, with significant increases in accumulation of the non-potyvirus partner, as in the case of melon plants infected by the potyvirus *Watermelon mosaic virus* (WMV) and the crinivirus *Cucurbit yellow stunting disorder virus* (CYSDV). To further explore the synergistic interaction between these two viruses, the activity of RNA silencing suppressors (RSSs) was addressed in transiently co-expressed combinations of heterologous viral products in *Nicotiana benthamiana* leaves. While the strong RSS activity of WMV Helper Component Proteinase (HCPro) was unaltered, including no evident additive effects observed when co-expressed with the weaker CYSDV P25, an unexpected negative effect of WMV P1 was found on the RSS activity of P25. Analysis of protein expression during the assays showed that the amount of P25 was not reduced when co-expressed with P1. The detrimental action of P1 on the activity of P25 was dose-dependent, and the subcellular localization of fluorescently labeled variants of P1 and P25 when transiently co-expressed showed coincidences both in nucleus and cytoplasm. Also, immunoprecipitation experiments showed interaction of tagged versions of the two proteins. This novel interaction, not previously described in other combinations of potyviruses and criniviruses, might play a role in modulating the complexities of the response to multiple viral infections in susceptible plants.

## Introduction

The simultaneous presence of two unrelated viruses in mixed-infected plants can lead to different outcomes, including synergisms, antagonisms, and neutral interactions (Syller, 2012; Syller and Grupa, 2016; Moreno and López-Moya, 2020). Despite being a situation common in natural conditions, our knowledge of the interactions taking place in mixed infections is still rather limited, even for those combinations that cause plant diseases with more than one etiological viral agent. Particularly unknown is how the virus-virus interactions could influence pathogenicity and condition the ecology and evolution of viruses, even resulting in generation of new variants or shaping the genetic structure of viral populations (Tollenaere et al., 2016; Alcaide et al., 2020). Hence, a better knowledge of virus-virus interactions during mixed infections might be valuable for deploying efficient and durable virus control strategies (Syller, 2012; Wu et al., 2019).

Although the outcome of a mixed infection is difficult to predict in general, synergistic interactions are often expected when one of the partners is a potyvirus, assuming that the other unrelated virus would be “assisted” by the potyvirus. Initially characterized for the potyvirus *Potato virus Y* and the potexvirus *Potato virus X* (Damirdagh and Ross, 1967; Vance, 1991), the interactions of potyviruses and unrelated viruses produced outcomes remarkably coincidental in many cases (Taiwo et al., 2007; Zeng et al., 2007; Mascia et al., 2010). The identification of the potyviral Helper Component Proteinase (HCPro) as a candidate RNA silencing suppressor (RSS) (Anandalakshmi et al., 1998; Kasschau and Carrington, 1998) provided a sort of mechanistic model to explain the outcome, but the multifunctional nature of HCPro, including the complexities of its activity as RSS (Lakatos et al., 2006; Valli et al., 2018) makes specially challenging to reveal the underlying molecular aspects. Most of the interactions involving a potyvirus and an unrelated virus have been described only partially, mainly attending to the macroscopic and visible outcomes, likely leaving many unexplored molecular mechanisms. Indeed, the simplistic view in which a potent RSS would always work in a pro-viral direction for other viruses might not respond to the underlying complexities of these interactions. Interestingly, one remarkable exception to the potyvirus-assisted synergistic interactions was reported in sweet potato crops, where the potyvirus partners were benefited in co-infections with the crinivirus *Sweet potato chlorotic stunt virus* (SPCSV; Tairo et al., 2005; Untiveros et al., 2007; Clark et al., 2012). For this exception, a gene product different of HCPro, the P1N-PISPO, was associated to RSS activity in the potyvirus *Sweet potato feathery mottle virus* (SPFMV; Mingot et al., 2016; Untiveros et al., 2016).

In the present work, we have considered the mixed infection of the potyvirus *Watermelon mosaic virus* (WMV) and the crinivirus *Cucurbit yellow stunting disorder virus* (CYSDV). These two viruses belong to different taxonomic families, *Potyviridae* (Wylie et al., 2017) and *Closteroviridae* (Fuchs et al., 2020), they are transmitted by different vectors, but they are commonly found together in melon and other cucurbits, causing high production losses (Juarez et al., 2013). WMV is a widely spread aphid-transmitted potyvirus with the usual genomic and biological characteristics of the genus (Revers and García, 2015; Valli et al., 2015; Gibbs et al., 2020), and a remarkable natural variability (Moreno et al., 2004; Desbiez et al., 2011; Verma et al., 2020). CYSDV is a whitefly-transmitted crinivirus (Abrahamian and Abou-Jawdah, 2014; Wintermantel et al., 2017), and less widespread than WMV although lately it is becoming an emergent problem together with other whitefly-transmitted viruses (Navas-Castillo et al., 2011, 2014). We have already characterized the dynamic accumulation of these two partner viruses in melon and explored how that influences their vector-mediated dissemination (Domingo-Calap et al., 2020), although many molecular details of their interaction remained unaddressed, such as those dealing with RNA silencing processes.

To infect a host plant, viruses need to counteract its RNA silencing mechanism, considered an innate immune response (Voinnet, 2001; Baulcombe, 2004), by producing suppressor proteins that target different steps of the pathway to block this antiviral response (Csorba et al., 2015). In CYSDV, the role of RSS is associated to P25 (Kataya et al., 2009), and in WMV, we hypothesized that it might reside in HCPro, as this is the most common RSS in many other potyviruses (Valli et al., 2018). This assumption about WMV HCPro acting as RSS was confirmed experimentally for the first time in the present study.

To further explore virus-virus interactions in mixed infections of WMV and CYSDV, we decided to focus on their RNA silencing suppression machinery. In addition to the known RSSs, other viral gene products that might participate in the activity have been selected. For instance, the rather variable P1 of potyviruses (Valli et al., 2007; Shan et al., 2015) that has been considered a modulator of RSS in several viruses (Fernández et al., 2013; Pasin et al., 2014) and the P22 of CYSDV, the gene product downstream of the P25 region in the RNA1, located in a position where other criniviruses encode proteins involved in this function (Kreuze et al., 2005; Cañizares et al., 2008; Weinheimer et al., 2015; Kubota and Ng, 2016; Chen et al., 2019; Orfanidou et al., 2019).

In this study, thematically independent of our previous publication on the same mixed infection of WMV and CYSDV in melon (Domingo-Calap et al., 2020), we report an unexpected and dose-dependent negative effect of WMV P1 on the RNA silencing suppression activity of CYSDV P25 when co-expressed in a transient assay in *Nicotiana benthamiana*, and discuss its possible contribution to the complex virus-virus interactions during mixed infections.

## Materials and Methods

### Plasmid Constructs

Gene fragments corresponding to CYSDV P25 (639 nts), CYSDV P22 (579 nts), WMV P1 (1,332 nts), and WMC HCPro (1,371 nts) were RT-PCR-amplified using viral genomes extracted respectively from CYSDV (for P25 and P22) and WMV (for P1 and HCPro) infected plants, using Phusion High Fidelity PCR System (Thermo Sciences) and the specific primers shown in Table 1. A cis construct spanning P1HCPro was also prepared using primers forward and reverse upstream P1 and downstream HCPro, respectively. The amplified PCR products were purified and cloned into pENTRY D-TOPO *GATEWAY* expression system (Invitrogen), resulting in the constructs pENTRY_CYSDV-P22, pENTRY_CYSDV-P25, pENTRY_WMV-P1, pENTRY_WMV-HCPro, and pENTRY_WMV-P1HCPro. Subsequently, the different viral genes were mobilized through LR recombination into the different destination plasmid vectors (Tanaka et al., 2011), including pGWB-702 (containing the 35S promoter and the *Ω* enhancer) for silencing suppression analysis; pGWB-742 (containing 35S promoter and N-terminal phusion to EYFP) and pGWB-745 (same promoter and N-terminal phusion to ECFP) for subcellular localization; pGWB 715 (providing N-terminal tag 3xHa) and pGWB 718 (providing N-terminal tag 4xMyc) for protein detection and co-immunoprecipitation assays. For bimolecular fluorescence complementation (BiFC) assays (see below), the destination plasmid vectors pBiFC2 and pBiFC3 were used (Azimzadeh et al., 2008; Ochoa et al., 2019).

**Table 1:** Sequence of primers used for cloning viral gene products.

Gene product	Sense^1^	Primer sequence^2^
WMV P1	Fw	5' **CACC**ATGGCAACAATCATGTTTGGAG 3'
	Rv	5' TCAATAATGTTGAATATCTTCTATCTCC 3'
WMV HCPro	Fw	5' **CACC**ATGTCTCACACTCCAGAAG 3'
	Rv	5' TCAACCAACCCTGTAAAACTTC 3'
CYSDV P22	Fw	5' **CACC**ATGCAGAGTGTTGGAGTAG 3'
	Rv	5' TCAAGGGATGGTGCCCATG 3'
CYSDV P25	Fw	5' **CACC**ATGGGAGAAGATTTACAAGAAC 3'
	Rv	5' CTACTCCAACACTCTGCATTC 3'

1 Sequence corresponding to the viral genome are considered Forward (Fw), while complementary are Reverse (Rv).

2 Bold nucleotides correspond to 5' additions required for properly oriented cloning in pENTR-TOPO. In the case of potyviral gene products, a methionine codon inserted in the forward primer for Helper Component Proteinase (HCPro) and sequences complementary to stop codons added in the reverse dowstream primers of both P1 and HCPro are underlined.

### Agroinfiltration and Green Fluorescent Protein Imaging

*Nicotiana benthamiana* plants were grown at 23–25°C with a photoperiod of 16 h of light and 8 h of darkness. Cultures of *Agrobacterium tumefaciens* strain EHA105 carrying the different plasmids were grown overnight at 28°C, and cells were resuspended to an equal OD_600_ (=0.3) in induction buffer (10 mM MES/NaOH, pH 5.6, 10 mM MgCl_2_, 150 μM acetosyringone) for 3 h before agroinfiltration of patches in leaves of *N. benthamiana* plants at the 4–6-leaf growth stage. For co-infiltration, the *A. tumefaciens* cultures were adjusted to the same optical density at OD_600_ (=0.3) and mixed in induction buffer to be agroinfiltrated at the same time.

The identification of RNA silencing suppression activity was done by visual inspection of green fluorescent protein (GFP) fluorescence in agroinfiltrated leaves, comparing the different independent viral proteins P22, P25, P1, HCPro, and the cis construct P1-HCPro when expressed transiently (see above) through co-agroinfiltrated with the construct pBIN-GFP, using always in every leaf for comparison purposes both a positive (corresponding to the CVYV P1b RSS) and negative (an empty vector named delta) controls kindly provided by Dr. A. Valli (CNB-CSIC, Madrid, Spain), and essentially following previously described procedures (Giner et al., 2010; Mingot et al., 2016). For the combinations of different constructs, the OD was adjusted to keep equal concentration of bacteria in the agroinfiltration solution. GFP fluorescence was observed under long-wavelength UV light (Black Ray model B 100AP, UV products), and pictures were taken using a Nikon digital camera.

### Quantitative RT-PCR

Total RNA was extracted from two leaf disks of agroinfiltrated *N. benthamiana* plants using TRIzol reagent (Invitrogen) according to the provider’s instructions, including an additional ethanol precipitation step to improve purity of RNA. Quality and concentration of RNA was estimated using a NanoDrop® spectrophotometer (ND-8000). After DNase treatment to eliminate genomic DNA, about 1 μg of total RNA extracted from plant samples was used to produce cDNA with the High-Capacity cDNA Reverse Transcription kit (Applied Biosystems™), following protocols provided by the manufacturer. SYBRGreen (Roche) was used to detect PCR products in a Light Cycler 480 (Roche) equipment using triplicates of 100 ng of the resulting single-stranded cDNA. Specific primers previously described for GFP (Leckie and Neal Stewart, 2011) and ubiquitin (Lacomme et al., 2003) sequences were used. Statistical analysis was performed applying *t*-test to ∆Ct values using the program GraphPad Prism version 6.0.

### Protein Extract Preparations and Western Blotting

Tagged versions of the different viral gene products were constructed using pGW 715 and pGW 718 backbones, and mobilized to *A. tumefaciens* for agroinfiltration in *N. benthamiana* (see above). Samples (four leaf disks) collected were processed from mock or agroinfiltrated *N. benthamiana* plants were collected and homogenized in 200 ul of extraction buffer (20 mM Tris-HCl pH 7.5, 30 mM NaCl, 1 mM EDTA, 0.5% NP-40, 2% b-mercaptoethanol). Cell debris were removed by centrifugation at 13,200 rpm at 4°C for 10 min, and an aliquot (30 μl) of the supernatant were boiled in Laemmli’s sample buffer (250 mM Tris-HCl pH 7.5, 40% Glicerol, 8% SDS, 20% b-mercaptoethanol). Samples were separated on 12% SDS-PAGE, transferred to Amersham Protran nitrocellulose blotting membrane and subjected to Western blot analysis. For detection, Anti-Myc Tag Antibody, clone 4A6 (Millipore) and Anti-Ha (Sigma-Aldrich) were used followed by incubation with adequate secondary anti-mouse antibodies. The proteins were visualized by chemiluminescence (Super Signal West Femto, Thermo Scientifics) according to the manufacturer’s instructions using a ChemiDoc imaging system (BioRad).

### Subcellular Localization and Co-localization

The coding gene products for WMV P1 and CYSDV P25 proteins were inserted in the vectors pGWB742 (35S pro, N-EYFP) and pGWB745 (35S pro, N-ECFP) respectively, generating constructs for expression of YFP-WMV-P1 and CFP-CYSDV-P25 fusion proteins. The transient expression of both products in *N. benthamiana* leaves was achieved co-agroinfiltrating the constructs with an additional plasmid for expression of the RSS P19 of TBSV, pBin-TBSV-P19 (kindly provided by Dr. Montse Martin, CRAG, Barcelona, Spain). A confocal laser scanning Leica TCS SP5 (Leica Microsystems, Germany) microscope was used to observe the *N. benthamiana* epidermal cells at the adequate wavelengths for each reporter.

### BiFC Assays

Expression of fusion proteins with N- and C-fragments of the reporter YFP, denominated, respectively YFP^N^ and YFP^C^ was achieved in *A. tumefaciens* strain EHA105 carrying plasmid for YFP^C^-WMV-P1, YFP^N^-WMV-P1, YFP^C^-CYSDV-P25, and YFP^N^-CYSDV-P25. Each construct and pBIN-TBSV-P19 were cultured separately and the cells were resuspended to an equal OD_600_ (=0.3). Equal volumes of the combinations YFP^C^- YFP^N^- and pBIN-TBSV-P19 were mixed in induction buffer (10 mm MES/NaOH, pH 5.6, 10 mm MgCl_2_, 150 μm acetosyringone). *Nicotiana benthamiana* plants at the 4–6-leaf stage were used for agroinfiltration. At 3, 5, and 7 days post agroinfiltration (dpa), epidermal cells of agroinfiltrated leaves were observed for fluorescence emission under a confocal laser scanning microscope (Leica TCS SP5) at a wavelength of 514 nm.

### Co-immunoprecipitation of Tagged Proteins

For immunoprecipitation 50 ul of Anti-c-Myc agarose beads (Sigma) were washed before adding the samples with Phosphate-Buffered Saline (PBS) 1x. Samples (about 1 g) of mock-, HA-WMV-P1, MYC-CYSDV-P25, or HA-WMV-P1 + MYC-CYSDV-P25 agroinfiltrated *N*. *benthamiana* leaves were collected, ground in immunoprecipitation buffer (20 mM Tris-HCl pH 7.5, 30 mM NaCl, 1 mM EDTA, 0,5% NP-40, 2% b-mercaptoethanol) and cleared by centrifugation at 13,200 rpm for 10 min at 4°C. Supernatants of the different lysates were added to samples of washed beads and incubated for 1 h at 4°C. After immunoprecipitation, beads were washed three times with ice-cold PBS 1x for 1 min each. Input extracts and eluates of immunoprecipitations were used for Western blot analysis (see above).

## Results

### Confirmation of RNA Silencing Suppression Activity for Transiently Expressed Viral Gene Products

**Figure 1:**
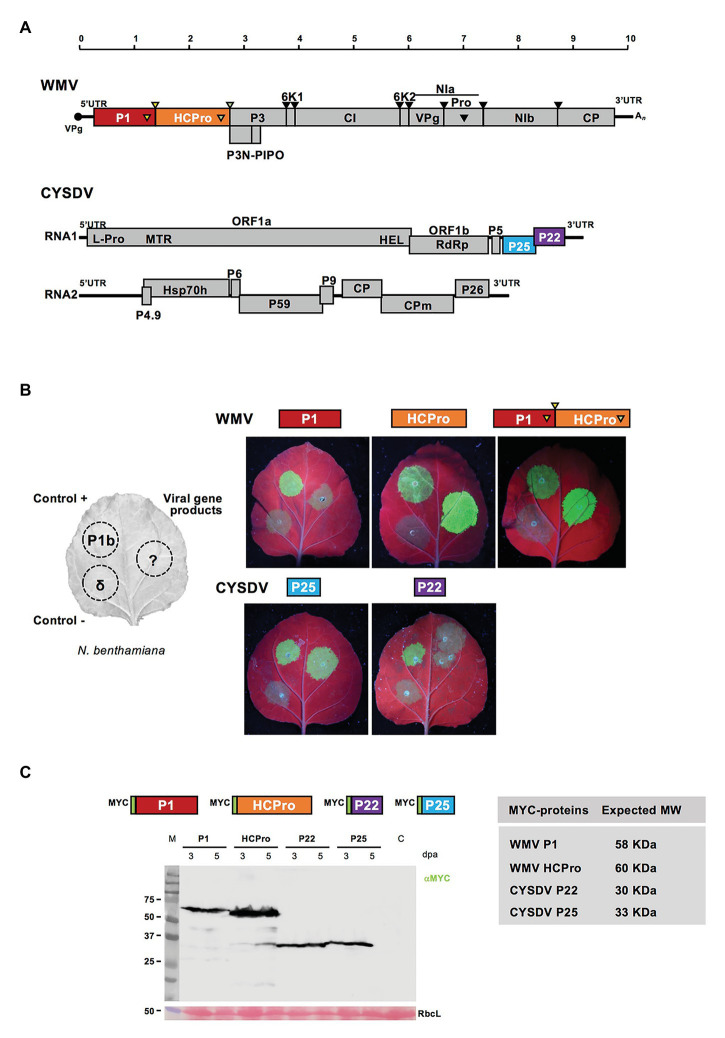
Confirmation of RNA silencing suppression (RSS) activity in individually expressed viral gene products of *Watermelon mosaic virus* (WMV) and *Cucurbit yellow stunting disease virus* (CYSDV). **(A)** Genome maps of WMV and CYSDV. Below the size rule in kilobases, the viral ssRNAs are shown as solid horizontal lines (10,035 nucleotides for WMV, and 9,123 and 7,976 for the RNA1 and RNA2 of the bipartite CYSDV, respectively). In the WMV genome, VPg is depicted as a solid circle at the 5' end, and the poly-A tail as An at the 3' end. Viral ORFs are depicted as boxes with the names of the mature gene products. The PIPO region is shown below the polyprotein of the potyvirus leading to the partially out-of-frame product P3N-PIPO, and the protease-specific cleavages sites are indicated by arrows above and matching symbols in the gene products responsible of the proteolytic process. The different frames are shown for the crinivirus gene products. **(B)** The left part of the panel shows schematically the organization of patches in the *Nicotiana benthamiana* leaves used to test RSS activity in co-agroinfiltration of the selected gene products with the reporter green fluorescent protein (GFP). The positions for positive and negative controls, corresponding to the P1b of *Cucumber vein yellowing virus* (CVYV) and an empty vector (delta), respectively, are also shown. Constructs for expression of the individual gene products and the P1-HCPro cis construct are indicated above the pictures of leaves. Pictures were taken at 5 days post agroinfiltration (dpa) under UV light. **(C)** A representative Western blot analysis of the N-terminus MYC-tagged gene products shown in the diagrams with their expected molecular weights shown in the table. A representative blot revealed after incubation with the indicated anti-MYC specific antibody and the corresponding anti-mouse, is shown with agroinfiltrated samples, collected at 3 and 5 dpa time points, as indicated, and a non-agroinfiltrated *N. benthamiana* control lane labeled as C. M lane shows the migration of pre-stained molecular weight markers (sizes in KDa on the left side). RbcL corresponds to the Ponceau red-stained blot showing the large subunit of Rubisco protein as loading control.

Individual gene products from the viruses WMV and CYSDV were selected for testing their activities, including already known RSSs and others with potential modulator effects. For the potyvirus, P1 and HCPro proteins were tested, and in the crinivirus, we chose P25 and P22, both located in RNA1 (Figure 1A). The different constructs were tested individually using the standard transient expression assay in *N. benthamiana* leaves with GFP as reporter, and the corresponding CVYV P1b and delta constructs as positive and negative controls, following described procedures (Giner et al., 2010; Mingot et al., 2016). In the experiments with individual gene products, both WMV HCPro and CYSDV P25 exhibited activities as RSSs at 3 and 5 dpa, lasting up to at least 7 dpa in the case of HCPro, while P1 and P22 did not show detectable GFP at any of the tested time points, indicating that they do not suppress local RNA silencing in the assay (Figure 1B). These results served to confirm the RSS activity of the P25 protein in our CYSDV Spanish isolate, and to visually determine for the first time the RSS activity of the HCPro protein of WMV, as it was expected attending to the antecedents for many other viruses in the same genus (Valli et al., 2018). Since P1 and HCpro are naturally expressed in cis as part of a larger polyprotein, the construct P1-HCPro with the two gene products in cis was also tested, showing again a strong RSS activity, indistinguishable of the activity exhibited by the WMV HCPro alone (Figure 1B).

To verify the correct expression of all viral products constructs, MYC-tagged versions were agroinfiltrated and samples analyzed by SDS-PAGE and Western blot (Figure 1C).

### Combination of Heterologous Gene Products: Negative Effect of WMV P1 on the RSS Activity of CYSDV P25

**Figure 2:**
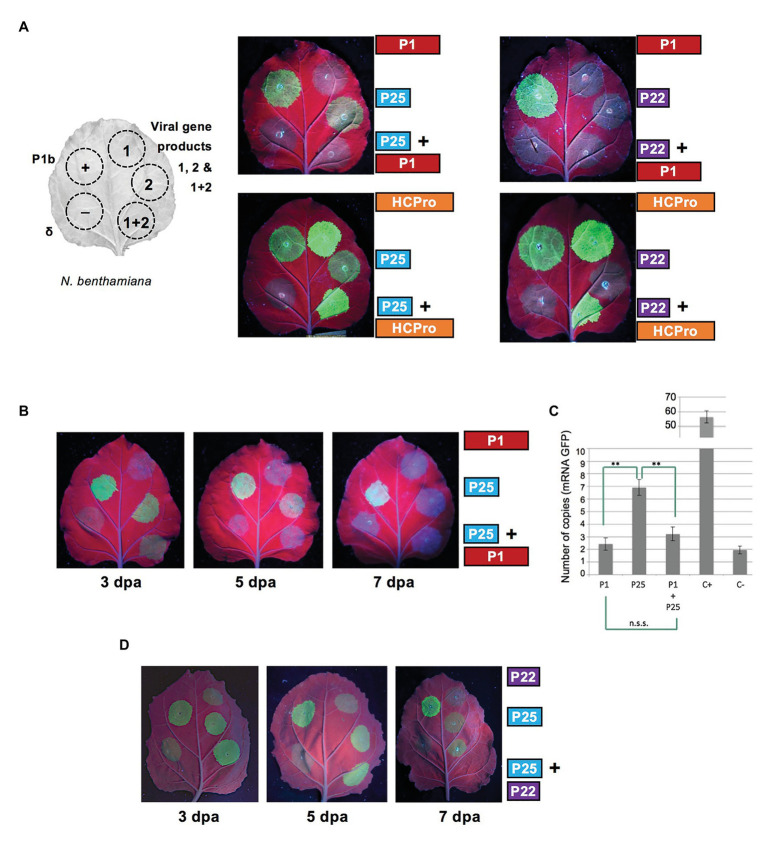
Effect on RSS activity of the combination of heterologous selected gene products of WMV and CYSDV. **(A)** Schematic organization of patches in *N. benthamiana* leaves and comparison of effects on RSS activity of individual and combined gene products when co-agroinfiltrated with the reporter GFP. The positions for positive and negative controls, corresponding to the P1b of CVYV and an empty vector (delta), respectively, are also shown in the left half of every leaf. In the right side of the pictures and adjacent to the patches are depicted the constructs for expression of the individual gene products of WMV (P1 or HCPro) and of CYSDV (P25 or P22), with their corresponding combinations in the lower row. Pictures were taken at 5 dpa under UV light. **(B)** Time course evolution of RSS activity at 3, 5, and 7 dpa for the individual gene products WMV P1 (upper right patches), CYSDV P25 (central right patches), and their combination (lower right patches). Positive and negative controls as in A (patches in left side halves). **(C)** Quantification of GFP mRNA by qRT-PCR, relative to the reference gene ubiquitin, at 3 dpa, in patches agroinfiltrated with the constructs indicated below the bars. Mean values and SDs of three independent replicates are plotted, indicating statistically significant differences after *t*-test analysis (**indicate *p* < 0.05, values of *p* = 0.0013 for P25 vs. P1, and *p* = 0.0022 for P25 vs. P1+P25). **(D)** Absence of effect on RSS activity of CYSDV P25 when co-agroinfiltrated with CYSDV P22. Positive and negative controls as in Figure 1B (patches in left side half). Pictures were taken at 3, 5, and 7 dpa under UV light.

To determine possible interactions between gene products of the two viruses considered, a set of experiments were designed to compare the performance in assays of RSS activity of different combinations involving WMV P1 or HCPro, paired together with CYSDV P22 or P25 (Figure 2A). In the combinations including WMV HCPro as one of the partners, the high RSS activity remained apparently unaltered when comparing in the same leaf patches infiltrated with HCPro alone or co-expressed along with either P22 or P25, suggesting that the assay was not sensible enough to detect an additive effect of P25 on top of the very high activity exhibited by HCPro. However, in the reciprocal combinations involving WMV P1, while its expression in the control with P22 remained non-functional as expected, a negative effect of the non-suppressor WMV P1 was observed on the RSS activity of the CYSDV P25 protein. This unexpected result was consistently reproduced, always showing an obvious attenuation of the intensity of the GFP signal with respect to that observed when the suppressor was expressed alone (a representative example is shown in the upper left photograph of Figure 2A). The same effect could be observed along different time points during the experiment, as shown for 3 and 5 dpa, before the weak activity of P25 faded after 7 dpa (Figure 2B). To further confirm these observations, we performed relative qRT-PCR measuring the expression levels of the GFP mRNA (Figure 2C), showing that the negative effect was present as early as at 3 dpa, when visually only a weak RSS activity was observed in the patches co-agroinfiltrated with P1+P25 (Figure 2B, see below).

To rule out an unspecific effect on CYSDV P25 caused by co-expression of any other protein, we tested as an additional control if the co-expression of CYSDV P22 could also affect the RSS activity of P25. The experiment was performed as described above, finding that the capacity of P25 to exhibit RSS activity was unaltered by the co-expression of P22 at the same 3 and 5 dpa time points (Figure 2D).

### Dynamics of Protein Expression in Patches Agroinfiltrated With CYSDV P25 and WMV P1, Both Individually and in Combination

**Figure 3:**
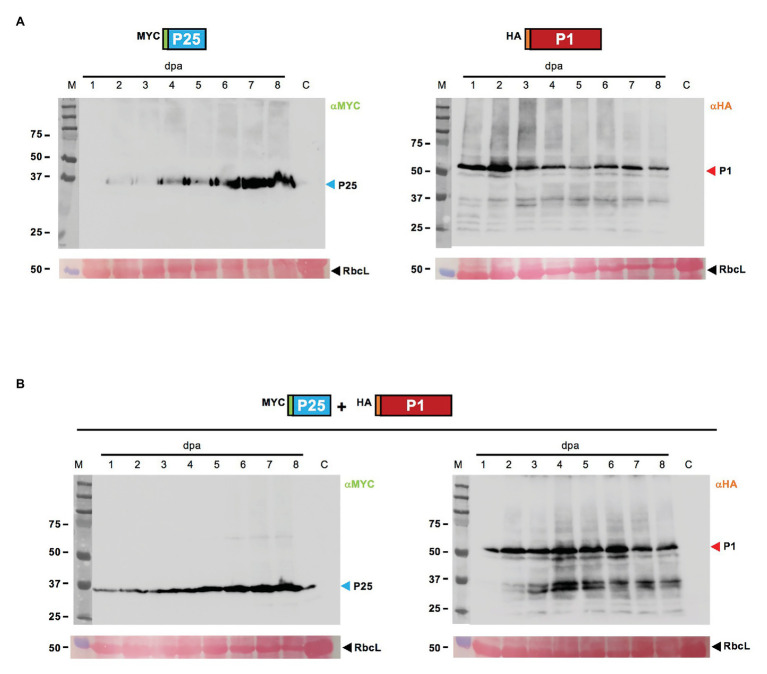
Western blot analysis of selected N-terminus tagged proteins after transient expression, individually or combined. **(A)** Samples of *N. benthamiana* patches individually agroinfiltrated with MYC-P25 (left panel) and HA-P1 (right panel) constructs collected daily between 1 and 8 dpa are shown, besides a non-agroinfiltrated *N. benthamiana* control lane labeled as C. The blots are revealed after incubation with the corresponding anti-MYC or anti-HA specific antibodies. **(B)** Samples of patches co-agroinfiltrated with MYC-P25 and HA-P1 collected daily between 1 and 8 dpa are shown, besides a *N. benthamiana* control lane labeled as C, and revealed with anti-MYC (left panel) or anti-HA (right panel) specific antibodies. For both **(A,B)** lanes labeled with M show the migration of pre-stained molecular weight marker (sizes in KDa on the left side), and RbcL correspond to the Ponceau red-stained blots showing the large subunit of Rubisco protein as loading control.

Epitope tagged versions of the two gene products were tested for expression after agroinfiltration. Two different tags, MYC in the case of CYSDV P25 (MYC-P25) and HA in the case of WMV P1 (HA-P1), were chosen to allow independent detection of each gene product in the co-agroinfiltrated patches, and samples (pooled of three leaves from three independent plants) were taken daily up to 8 dpa. Representative Western blot analysis with the corresponding specific antibodies is shown in Figure 3. In the patches agroinfiltrated individually (Figure 3A), a steady increase of MYC-P25 expression was observed, probably reflecting its own RSS activity, reaching the highest amount of detectable protein at the end of the sampling period (8 dpa), while HA-P1 expression apparently peaked as early as 2 dpa, later showing a slight reduction followed by near constant levels until the last day sampled. In the case of the patches co-agroinfiltrated with MYC-P25 and HA-P1, the analysis also showed detection of both proteins along the complete period, with a delay in the peak of HA-P1 occurring around day 4, and a very similar dynamic of steady accumulation in the case of MYC-P25 (Figure 3B). These results proved that the reduced RSS activity of CYSDV P25 when co-expressed with WMV P1 was not caused by lack of expression.

### Dose-Dependent Effect of the Presence of WMV on the RSS Activity of CYSDV P25

**Figure 4:**
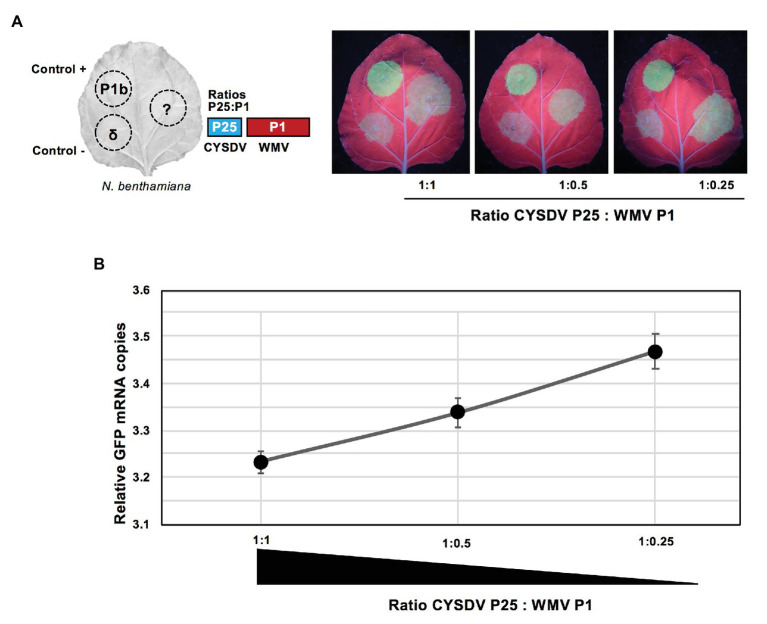
Dose response of the presence of WMV P1 on the RSS activity of CYSDV P25. **(A)** Schematic organization of patches in *N. benthamiana* leaves co-agroinfiltrated with the reporter GFP. The same fixed concentration of P25 is used together with different amounts of P1 to reach the indicated ratios as shown in each picture, with positive and negative controls included in the left half of every leaf as in Figure 1B. Pictures were taken at 3 dpa under UV light. **(B)** Quantitative values showing inverse correlation of the ratio of CYSDV P25: WMV P1 and the relative copies of GFP mRNA measured by qRT-PCR with ubiquitin as reference gene. The graph shows the values for average and SD corresponding to three biological replicates per treatment.

To evaluate if the observed negative effect was correlated to the relative expression levels of the two viral products, an experiment of dose-response was designed. Using the same concentration of *A. tumefaciens* culture transformed with the construct for expression of CYSDV P25, up to three dilutions of the culture harboring the partner product WMV P1 were tested for their effects on the RSS activity. Assuming that the quantities of CYSDV P25 were kept constant, the quantities of the partner WMV P1 were decreasing exponentially by a factor of 2, which correlated with a visible increase of GFP under UV light (Figure 4A), also detectable as a linear increase of mRNA levels corresponding to GFP (Figure 4B). These results indicated that the RSS activity of CYSDV P25 recovered when the relative amount of WMV P1 decreased.

### Subcellular Localization of WMV P1 and CYSDV P25 in Nucleus and Cytoplasm

**Figure 5:**
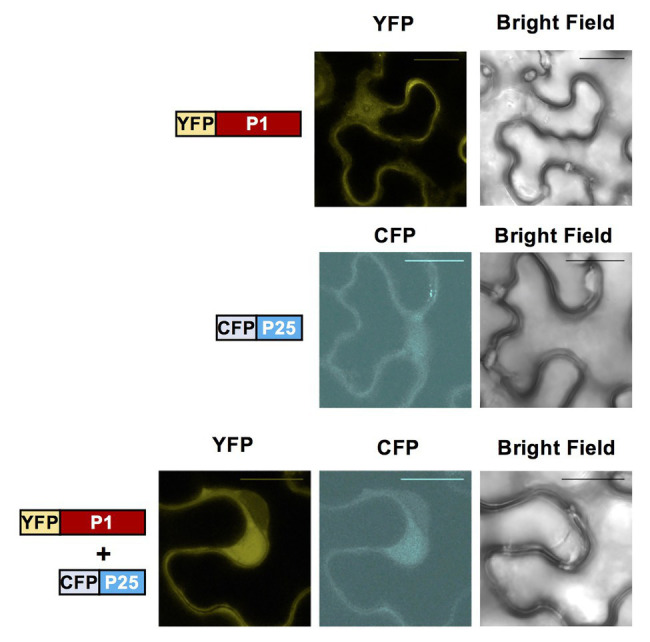
Subcellular localization of fluorescently labeled WMV P1 and CYSDV P25 proteins. Confocal laser microscopy of *N. benthamiana* leaves agroinfiltrated with the constructs indicated schematically on the left: P1-YFP (upper lane), P25-CFP (central lane) and with both constructs at the same time (bottom lane). The images labeled with YFP correspond to the yellow color field, those labeled with CFP to the blue color field, and in the last column the bright field is shown, the column. In the samples agroinfiltrated with individual constructs observations were also performed with the conditions for the two YFP and CFP, without detecting any cross fluorescence (not shown). Bar size 20 μm.

To investigate the subcellular localization of WMV P1 and CYSDV P25 proteins, we cloned them into constructs fused to fluorescent markers using the plasmids pGWB742 (containing YFP for fusion to the N-terminus of the cloned protein) and pGWB745 (for fusion to CFP, also in N-terminus). The constructs were transformed into *A. tumefaciens* strain EHA105 and agroinfiltrated into *N. benthamiana* leaves. Confocal microscopy examination showed that both fusion proteins, WMV P1 tagged with YFP and CYSDV P25 tagged with CFP, were located in the nucleus and the cytoplasm of the agroinfiltrated cells, and that they apparently co-localize in both compartments (Figure 5).

### Interaction of WMV P1 and CYSDV P25

**Figure 6:**
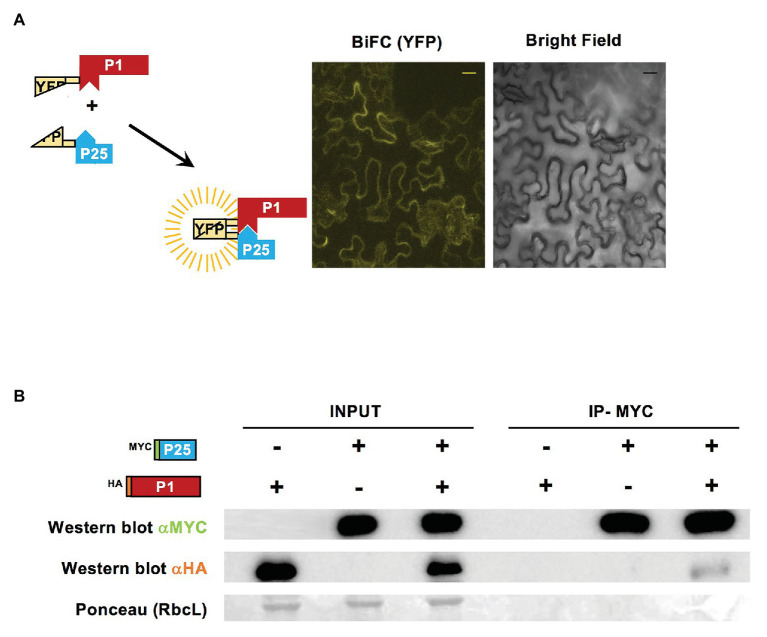
Interaction of WMV P1 and CYSDV P25. **(A)** Schematic representation of bimolecular fluorescence complementation (BiFC) constructs with the split YFP fused to WMV P1 and CYSDV P25, and the observation using confocal laser microscopy of *N. benthamiana* leaves agroinfiltrated simultaneously with the two constructs. The picture YFP corresponds to the yellow color field and the same area is shown under bright field illumination. Control samples agroinfiltrated with individual constructs were analyzed without detecting any fluorescence (not shown). Bar size 20 μm. **(B)** Immunoprecipitation of viral protein variants tagged with MYC or HA, as indicated. Western blot analysis with anti-MYC and anti-HA antibodies, and Ponceau staining of the large subunit of Rubisco (RbcL) as control of loading, are shown for the input fractions and the corresponding immunoprecipitation samples, as indicated.

To test if there was a direct interaction between WMV P1 and CYSDV P25 when co-expressed transiently in *N. benthamiana*, we performed BiFC and co-immunoprecipitation assays. As shown by confocal microscopy observations, the split YFP fragments fused to WMV P1 and CYSDV P25 did reconstitute a visible fluorescence with the appropriate filter, indicating that the two proteins could interact in the cytoplasm of the agroinfiltrated cells (Figure 6A).

Furthermore, when the same two tagged proteins with MYC and HA epitopes used in the previous time course analysis (see Figure 3) were co-agroinfiltrated and tested for co-inmunoprecipitation, a product corresponding to HA-P1 was precipitated along with the MYC-P25, indicating that indeed the two proteins can interact (Figure 6B).

## Discussion

Despite the common occurrence of multiple viral infections in plants, in many cases our understanding of the interactions occurring when two or more unrelated viruses share the same plant is still incomplete (Moreno and López-Moya, 2020). As an example, our recent analysis of melon plants co-infected by WMV and CYSDV revealed a complex scenario, with dynamic changes along the progress of the infections, that could even affect the transmissibility of the viruses by their insect vectors: briefly, the initial synergism and boost of CYSDV accumulation was later moderated and accompanied by a sort of recovery phenotype (Domingo-Calap et al., 2020). Intrigued by this peculiar behavior, we have addressed if virus-virus interactions between WMV and CYSDV might help to explain the outcome of the mixed infection. To start exploring at the molecular level the interactions between the two partner viruses, we decided to consider first the RSS function. Experiments were designed to transiently express the gene products known to participate in this function during individual infections, and then to combine them in order to find out possible interactions. In addition to known RSSs, we included other gene products that might modulate their activity in RNA silencing suppression. Following this strategy, an unexpected negative effect on the RSS activity of the P25 of CYSDV was observed when this viral product was expressed together with the P1 of WMV. To rule out a possible effect on the expression level of CYSDV P25 when co-expressed along with WMV P1, we have tested the amounts of tagged protein versions by Western blot analysis in agroinfiltrated patches. Compared with the individually expressed protein, we did not observed lack of expression in CYSDV P25 at different time points, therefore supporting a true affectation of the RSS activity. Interestingly, the dynamic of accumulation of tagged WMV P1 appeared to be altered in the co-agroinfiltrated samples, with a delayed peak compared with the individually expressed control. Unfortunately, the damage suffered after agroinfiltration precluded longer analysis, but it is tempting to speculate if these changes along this limited time might reflect somehow the peculiar dynamics mentioned to occur during mixed infections (Domingo-Calap et al., 2020).

To our knowledge, this is the first description of an interaction between viral gene products of two unrelated plant viruses that interfere on the RSS activity of one of them. Before speculating about the importance of this observation, a couple of previous considerations are cautionary needed: (i) the effect was observed in transient expression, not during viral infections; and (ii) it was occurring in a different plant of the natural common hosts where the two viruses might co-exist. Thus, we cannot assume directly that our observations after transient expression could reflect exactly what occurs during co-infection of the two viruses in a naturally infected cucurbit host. Indeed, the localization and behavior of the selected gene products expressed transiently in *N. benthamiana* might be quite different to what really happens during infections in cucurbits. In other words, could this negative interaction be occurring as well during WMV and CYSDV co-infection? Unfortunately, this question is difficult to address. First, adequate infectious clones of CYSDV that could be manipulated for tagging gene products in plants are not yet available, being only reported a version capable to replicate in protoplasts (Owen et al., 2016). We attempted to use this tool for whitefly transmission assays and to recreate mixed infections, unfortunately with no success (unpublished data). Another limitation to directly study mixed infections of the two viruses derives from difficulties to infect *N. benthamiana*, the model plant species, where the transient expression observations were performed: despite being a highly susceptible plant for many viruses, including isolates of WMV (Lecoq et al., 2011; Aragonés et al., 2019), CYSDV appears not to be able to infect and not even replicate in protoplasts of this species (Owen et al., 2016). The alternative approach to test the activity of P25 in susceptible cucurbits previously infected with WMV would require to knock-out the activity of WMV HCPro, a rather complex task, which could compromise the infectivity of WMV or modify its pathogeneicity, as suggested by previous mutagenesis and variability studies performed with other potyviruses (Torres-Barceló et al., 2008; Han et al., 2016).

Regarding localization in the plant, our knowledge on the distribution of WMV and CYSDV during mixed infections is also incomplete. Crinivirus are phloem-restricted viruses, and CYSDV distribution within the plant is particularly variable (Marco et al., 2003). A different crinivirus of cucurbits has been recently tagged with GFP (Wei et al., 2018), but unfortunately constructing a similar tool for CYSDV is not feasible nowadays, as already mentioned. On the other hand, there are no specific studies on the distribution of WMV within different tissues and cells in the plant, but comparing to other potyviruses it was expected to invade more cell types (Kogovsek et al., 2011). As a further complication, the distribution of viruses in mixed and individual infections could be different, as it was shown for instance in combinations of potyvirus and cucumovirus (Ryang et al., 2004; Mochizuki et al., 2016). Interestingly, in sweet potato plants co-infected by SPCSV and potyviruses, crinivirus components were detected outside the phloem, in contrast with its restricted phloem localization in single infections (Nome et al., 2007). Despite the lack of information about WMV, we can still make an educated guess, considering that the presence of potyviruses in phloem has been shown in certain cases (Rajamäki and Valkonen, 2003; Ion-Nagy et al., 2006). Also, another potyvirus of cucurbits, *Zucchini yellow mosaic virus* (ZYMV), showed a broad distribution in Zucchini when tagged with a visual marker (Majer et al., 2017). Although, at this point, we cannot provide evidence for the presence of the two viruses co-infecting the same cells, there are sufficient antecedents to make this possibility plausible. Further studies will be required to verify if indeed WMV and CYSDV might coincide in certain cells, and also if their distribution is altered or not during mixed infections.

With respect to intracellular localization, the P1 of potyviruses was found in the cytoplasm associated to other viral products (Lehto et al., 1998), and also trafficking to the nucleolus as recently reported (Martinez et al., 2014). Our results are compatible with these localizations. However, no information is available regarding intracellular localization of CYSDV P25, and only limited data are reported in other criniviruses and for other gene products, such as those involved in cell-to-cell movement (Qiao et al., 2018). Again, further investigations will be required to better understand if our observations of transiently expressed P25 reflect the intracellular localization of this viral protein during the virus infection.

The function(s) played by the P1 of potyviruses has remained elusive, and only recently some insights about its role(s) as modulator of essential activities during infection are being revealed. As a first important point to consider, the P1 is a remarkably variable product among potyviruses, what argues for its participation in host range determination, as it has been suggested by different authors (Valli et al., 2007; Revers and García, 2015; Cui and Wang, 2019; Nigam et al., 2019). Works with *Plum pox virus* (PPV) showed that P1 is involved in replication and pathogenicity (Maliogka et al., 2012; Pasin et al., 2014). Concerning RSS related functions, it has been proposed that P1 might stimulate the activity of HCPro (Anandalakshmi et al., 1998; Pruss et al., 2004; Rajamaki et al., 2005), but it is unclear if this stimulatory effect could be exerted on other RSSs, especially considering the importance of their expression in cis (Fernández et al., 2013). Interestingly, partial truncation of P1 in PPV revealed an antagonistic role for P1 in self-processing with a negative impact in local infection (Shan et al., 2018), but again it is uncertain if similar effects can be expected as well in other viruses. Our finding here might provide further clues to disentangle if the role(s) played by P1 are particularly relevant in the case of mixed infections. Indeed, although the interaction between CYSDV P25 and WMV P1 was revealed because it affected the RSS function of the crinivirus protein, the changes in the dynamic of accumulation of P1 observed in our western blot analysis might suggest an effect on the potyvirus infection.

It will be interesting to find out if this kind of interactions might occur as well in other combinations of potyviruses plus criniviruses. Many important crops are susceptible to criniviruses (Tzanetakis et al., 2013; Abrahamian and Abou-Jawdah, 2014; Fiallo-Olivé and Navas-Castillo, 2019; Ruiz Garcia and Janssen, 2020), and therefore co-infections with potyviruses are very likely to occur. Particularly intriguing could be the case of sweet potato, suffering strong synergism but in the opposite direction, with a boost of the potyviruses and other unrelated virus accumulation when co-infected by the crinivirus SPCSV (Untiveros et al., 2007; Cuellar et al., 2015). As mentioned, the RSS of sweet potato-infecting potyviruses appears to differ from the usual activity of HCPro, with the function shifted to a partially out-of-frame gene product P1N-PISPO produced after polymerase slippage (Mingot et al., 2016; Untiveros et al., 2016).

Finally, it should be noted that each one of the virus partners will be producing several proteins simultaneously during a mixed infection, at least 10 and 13 different mature products for the potyvirus and the crinivirus, respectively. Thus, our analysis testing only a few heterologous products in combination of two by two elements is just a first attempt to start exploring a presumably much richer landscape of interactions. As a novel observation, we hope our work will stimulate further research to better understand if these kind of interactions form part of the expected fine-tuning of RSS and other important functions during mixed infections, and how it can contribute to virus pathogenicity in the different situations.

## Data Availability Statement

The original contributions presented in the study are included in the article/supplementary material, further inquiries can be directed to the corresponding author.

## Author Contributions

MD-C, OC, AM, and JL-M designed the research, analyzed the data, composed figures, and wrote the manuscript. MD-C, OC, ME, AM, and JL-M performed the experiments. All authors contributed to the article and approved the submitted version.

### Conflict of Interest

The authors declare that the research was conducted in the absence of any commercial or financial relationships that could be construed as a potential conflict of interest.
